# Real-world data on utilization of neoadjuvant chemotherapy for muscle invasive bladder cancer: impact on surgical complications and oncological efficacy

**DOI:** 10.2340/1651-226X.2025.42052

**Published:** 2025-01-02

**Authors:** Hege S. Haugnes, Håkon Kjæve, Eivind Bjerkaas, Ragnhild Hellesnes, Line Hjelle, Magnus Larsen

**Affiliations:** a Institute of Clinical Medicine, UIT - The Arctic University, Tromsø, Norway; b Department of Oncology, University Hospital of North Norway, Tromsø, Norway; c General Practice, Storsteinnes, Norway; d UroNord, Urology in North Norway, Tromsø, Norway; e Department of Urology, University Hospital of North Norway, Tromsø, Norway

**Keywords:** Neoadjuvant chemotherapy, cisplatin, cystectomy, bladder cancer, muscle invasive, utilization, complications, efficacy

## Abstract

**Background and purpose:**

Recommended treatment of urothelial muscle-invasive bladder cancer (MIBC) is cisplatin-based neoadjuvant chemotherapy (NAC) followed by cystectomy, but there are challenges with low utilization of NAC. We aimed to evaluate the utilization of NAC, perioperative complications and oncological efficacy in a real-world setting.

**Patients and methods:**

All patients operated with radical cystectomy at the University Hospital of North Norway during 2011–2021 for MIBC were included. NAC consisted of three cycles of dose-dense methotrexate, vinblastine, doxorubicin and cisplatin (ddMVAC) every second week. Complications after cystectomy (Clavien-Dindo ≥ grade 3 within 30 days), histopathologic NAC response, cancer recurrence, relapse-free survival (RFS), overall survival (OS) and cause of death were reported.

**Results:**

We included 124 patients, median observation time of 4 years. Fifty-nine patients (48%) received NAC. Most common causes for not receiving NAC were age ≥ 75 years (*n* = 38; 31%), cardiovascular disease (*n* = 7; 5.6%), and reduced kidney function (*n* = 6; 4.8%). Overall 34 patients (27%) had a ≥ grade 3 complication. The 5-year actuarial OS rate was higher among patients treated with NAC than those without NAC (67% vs. 45%, *p* = 0.02). Among NAC-treated patients, 29 (49%) were downstaged to non-muscle invasive stage (≤pT1), and the 5-year actuarial RFS and OS were higher among patients with ≤pT1 in the post-cystectomy specimen than those with ≥ pT2 (92% vs. 35%, and 94% vs. 39%, both *p* < 0.001).

**Interpretation:**

The utilization of NAC was high in this real-world setting. Treatment with ddMVAC with achieved downstaging to ≤pT1 was associated with considerably improved RFS and OS.

## Introduction

Recommended treatment of urothelial muscle-invasive bladder cancer (MIBC) is cisplatin-based neoadjuvant chemotherapy (NAC) followed by radical cystectomy and lymph node dissection [1]. Adjuvant nivolumab or pembrolizumab increases disease-free survival for subgroups of patients; however, overall survival (OS) data are lacking [2, 3]. Although the addition of NAC to surgery has led to an 8% absolute improvement in 5-year OS [4], MIBC remains a highly lethal disease with a historically 5-year OS at 53% for patients treated with NAC plus surgery. Recently, updated data from the VESPER trial reported 5-year OS at 66% after up to six cycles of neoadjuvant dose-dense methotrexate, vinblastine, doxorubicin and cisplatin (ddMVAC) [5].

There are promising data on the combination of immunotherapy with NAC in the perioperative setting [6], and recently an OS benefit with this combination was reported in the phase III Niagara trial [7]. Nevertheless, the most important current effort in the handling of patients with MIBC is to increase the utilization of NAC. The addition of NAC to surgery was demonstrated to increase OS in a meta-analysis already in 2005 [8], but there have been challenges with low utilization of NAC worldwide. A recent meta-analysis showed an overall NAC utilization of 17%, with the highest proportion of NAC in Japan at 44% [9].

Previous studies have identified high age and comorbidity as reasons for not receiving NAC [10, 11], and a concern about increased surgical complication rates. An increased rate of 90-day readmissions and more frequent use of blood transfusions after NAC plus cystectomy versus cystectomy alone has been reported [12, 13]. However, there is a lack of data on perioperative complications from population-based studies with a high NAC utilization rate. Smoking is associated with a higher risk of post-cystectomy complications [14], and has a negative impact on treatment efficacy [15]. Other factors possibly related to perioperative complications and survival are body composition, for example sarcopenia and change in body mass index (BMI) [1, 16, 17].

The aims of this study were to evaluate (1) the feasibility and toxicity of NAC among patients treated with radical cystectomy, (2) perioperative complications and oncological efficacy according to NAC status and response to NAC, and with regard to treatment period, smoking status and body composition.

## Material and methods

### Patients and treatment

**Figure 1:**
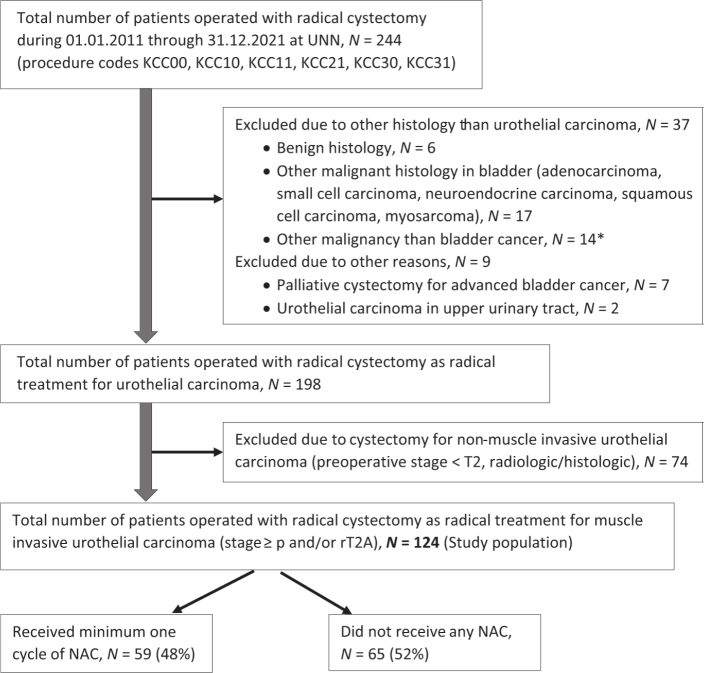
Flow chart for the study population. UNN: University Hospital of North Norway; N: number; NAC: neoadjuvant chemotherapy. *Patients operated with cystectomy as part of treatment for cancer in the prostate (*N* = 7), cancer in the colon (*N* = 4) or rectum (*N* = 3).

Overall 244 patients who underwent a cystectomy at The University Hospital of North Norway during January 1, 2011 through December 31, 2021 were identified through procedure codes in the medical record system. After excluding patients not eligible for this study (Figure 1), 124 patients operated with cystectomy as radical treatment for urothelial MIBC were included in the study population. Pre-treatment staging consisted of CT of the chest, abdomen and pelvis.

Neoadjuvant chemotherapy consisted of three cycles of ddMVAC given every second week if well-tolerated [18, 19]. Each cycle consisted of methotrexate 30 mg/m^2^ on day 1, and vinblastine 3 mg/m^2^, doxorubicin 30 mg/m^2^, and cisplatin 70 mg/m^2^ on day 2. The patients underwent a radiological evaluation with CT or MRI scan prior to the third cycle. In case of radiological progression, the third cycle was cancelled.

A radical cystectomy included removal of the bladder plus prostate and seminal vesicles in men, removal of the bladder plus uterus and adnexa in women, plus a pelvic lymph node dissection. A urinary diversion was always performed, most commonly as an ileac conduit. Surgery was performed as open surgery until the fall of 2019, after which robotic-assisted laparoscopic surgery was gradually implemented [20].

### Ethics declaration

The study was approved by the local data protection officer (project no. 02386).

### Assessments

Variables regarding patient characteristics, staging, and treatment of MIBC were collected retrospectively from medical records. Cancer recurrence status, survival, and cause of death was collected until April 2024. Treatment year was dichotomized into two categories (2011–2016 vs. 2017–2021), based on the gradual implementation of the national standardized cancer patient pathways in Norway during 2015 and 2016 [21]. Performance status was evaluated pre-chemotherapy among NAC treated patients according to the Eastern Cooperative Oncology Group (ECOG). Overweight was defined as BMI ≥ 25 kg/m^2^. Smoking status was dichotomized according to ever versus never smoker (daily or occasionally). Estimated glomerular filtration rate (eGFR) was calculated based on age and serum creatinine [22]. eGFR of 60–89 and 30–59 mL/min/1.73 m^2^ indicated a slightly or moderately decreased renal function, respectively [23].

Toxicity from chemotherapy was reported as a reason for discontinuing NAC. In addition, change in the proportion of patients with eGFR < 60 mL/min/1.73 m^2^ and relative weight change from the start of chemotherapy until cystectomy were calculated. Relative weight change was calculated as ((weight_cystectomy_–weight_chemotherapy_)/weight_chemotherapy_) × 100%, as described in a previous publication [24].

Surgical complications were quantified using the Clavien–Dindo classification system [25], a scoring system where complications are categorized from 1 to 5 on a severity scale, where category 5 indicates death. Only grade 3–5 complications within 30 days were reported, apart from infections where all grades were reported.

### Statistics

Continuous variables were presented as median (range), while categorical variables were presented as counts (proportion). Differences between groups were compared using Chi square test. Continuous variables (e.g. age) were compared using nonparametric tests (independent samples Median test). The observation time was calculated from the date of cystectomy until death or end of follow-up (as of April 2024).

Cumulative relapse-free survival (RFS) and OS were calculated using the Kaplan–Meier method. Survival curves for RFS and OS are presented according to NAC status, and according to response to NAC (non-muscle invasive; ≤pT1 vs. muscle-invasive; ≥pT2). Cox regression analyses assessed possible associations between different variables and RFS and OS, with time in years from cystectomy until relapse or death as the time scale. The following variables were tested in univariate analyses: age at cystectomy, treatment year, pT stage at cystectomy, treatment with NAC versus no NAC, smoking status, overweight, and kidney function. Variables with *p* < 0.10 and age at cystectomy were included in the multivariate Cox regression models. Inspection of minus log curves and assessment of Schonfeld residuals confirmed that the proportional hazard assumption was met for both RFS and OS analyses.

Statistical analyses were performed using the SPSS (version 29.0 SPSS Inc., Chicago IL) and STATA (version MP 18; STATA, College Station, TX) statistical software. Two-sided *p*-values <0.05 were considered significant.

## Results

### Chemotherapy utilization, toxicity and efficacy

Median age at cystectomy was 72 years (range 43–89) and median observation time was 4.0 years (range 0.0–13) (Table 1). Overall 59 patients (48%) received a minimum of one cycle of NAC. Among patients treated during 2011–2016 and 2017–2021, 45 and 51% received NAC, respectively (*p* = 0.59). The majority of NAC treated patients had ECOG 0 pre-chemotherapy (*n* = 48, 81%), while 10 (17%) and 1 (1.7%) had ECOG 1 or 2, respectively. Patients who received NAC were younger than those who did not receive NAC (median age 69 years vs. 77 years, *p* < 0.001). The most common causes for not receiving NAC were age ≥ 75 years (*n* = 38; 31%), cardiovascular disease (*n* = 7; 5.6%), and reduced kidney function (*n* = 6; 4.8%).

**Table 1:** Patient characteristics at the time of radical cystectomy for patients with urothelial MIBC, according to NAC versus no NAC and for all included patients.

Characteristic	NAC *N* = 59	No NAC *N* = 65	Total *N* = 124
Age, years	69 (42–79)	77 (47–89)	72 (43–89)
Observation time, years	5.2 (0.0–11.9)	3.4 (0.0–13)	4.0 (0.0–13)
Treatment year			
2011–2016	30 (45)	37 (55)	67 (54)
2017–2021	29 (51)	28 (49)	57 (46)
Male gender, *n* (%)	50 (84.7)	51 (78.5)	101 (81.5)
Smoking status, *n* (%)			
Never smoker	9 (15)	16 (25)	25 (20)
Ever smoker	50 (85)	49 (75)	99 (51)
BMI, kg/m^2^	24.9 (19.2–37.0)	25.0 (16.7–35.7)	24.9 (16.7–37.0)
BMI < 25 kg/m^2^, *n* (%)	32 (54)	32 (49)	64 (52)
eGFR, mL/min/1.73 m^2,a^	71 (29–110)	60 (26–107)	65 (26–110)
eGFR, categories, *n* (%)^a^			
eGFR < 60 mL/min/1.73 m^2^	16 (27)	31 (48)	47 (38)
eGFR 60–89 mL/min/1.73 m^2^	35 (59)	24 (37)	59 (48)
eGFR ≥ 90 mL/min/1.73 m^2^	8 (14)	10 (15)	18 (14)
Type of surgery, *n* (%)			
Open cystectomy	48 (81)	52 (80)	100 (81)
Robot-assisted	11 (19)	13 (20)	24 (19)
Ward time after cystectomy, days	13 (5–40)	14 (1–157)	14 (1–157)
cT stage before cystectomy, *n* (%)^b^			
T2	50 (86)	57 (88)	107 (87)
T3	7 (12)	5 (8)	12 (10)
T4	2 (2)	3 (5)	5 (3)
rN stage before cystectomy, *n* (%)^c^			
rN0	54 (92)	60 (92)	114 (92)
rN1	5 (8)	5 (8)	10 (8)
pT stage after cystectomy, *n* (%)			
pT0	17 (29)	2 (3)	19 (15)
pTa/PTis	10 (17)	2 (3)	12 (10)
pT1	2 (3)	4 (6)	6 (5)
pT2	15 (25)	20 (31)	35 (28)
pT3	14 (24)	28 (43)	42 (34)
pT4	1 (2)	9 (14)	10 (8)
pN stage after cystectomy, *n* (%)			
pNo	45 (76)	29 (45)	74 (60)
pN1	5 (8)	11 (17)	16 (13)
pN2	4 (7)	6 (9)	10 (8)
pNX	5 (8)	19 (29)	24 (19)

Data are presented as median (range) unless otherwise specified. There are no missing data.

MIBC: muscle-invasive bladder cancer; NAC: neoadjuvant chemotherapy; n, number; BMI: body mass index; eGFR: estimated glomerular filtration rate; c, clinical; r, radiological; p, pathological.

a eGFR is calculated based on age and serum creatinine [22].

b cT stage is based on either pathologic or radiologic T stage, where the highest T stage is presented, since this stage gave the indication for NAC. Among patients classified as cT2, 86 (80%) had pT2 stage, while 8 had pT1 stage and were upgraded due to radiologic T2 or T3 tumor.

c One patient treated with NAC had rNX stage.

Thirty-three patients (56%) received all three cycles of NAC. Twelve patients (20%) discontinued NAC after one cycle due to kidney failure (*n* = 6), infection (*n* = 2), and other causes. Additional 14 patients (24%) stopped NAC after two cycles (due to infection, kidney failure, patient decision, severe nausea, radiologic progression). NAC treated patients underwent cystectomy at a median of 4 weeks after day 1 in the last chemotherapy cycle (range 2–8 weeks).

The proportion of NAC treated patients with eGFR < 60 mL/min/1.73 m^2^ increased from 14% (*n* = 8) at start chemotherapy to 27% (*n* = 16) at cystectomy (*p* = 0.015). Overall 36 patients (61%) had stable weight or small to moderate weight gain. Eight patients (14%) had a small weight loss, while 15 (25%) had a moderate weight loss (> – 5%).

**Figure 2:**
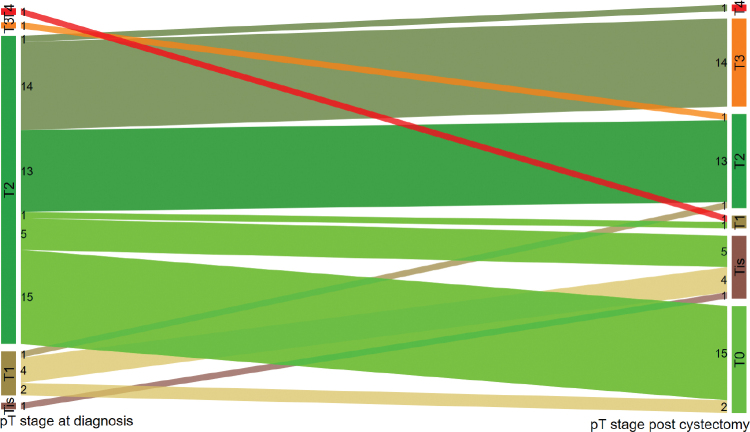
Figure 2 shows the pT stage at diagnosis (before chemotherapy) to the left, and pT stage post cystectomy to the right for NAC-treated patients. Numbers are the actual numbers of patients in each stage category.

Histopathologic evaluation of the cystectomy specimen showed that 29 patients (49%) had a downgrading of pT stage after NAC (Figure 2). Twenty-nine patients (49%) were downstaged to ≤pT1, of whom 17 (29%) were downstaged to pT0.

### Surgical complications

Overall 55 patients (53%) had a 30-day postoperative infection (Table 2). The infection rate was higher among patients aged ≥ 75 years than among those < 75 years (68% vs. 46%, *p* = 0.02), while there were no differences in infection rates for treatment period (*p* = 0.21), NAC status (*p* = 0.15), smoking status (*p* = 0.82) or BMI (*p* = 0.75).

**Table 2:** 30-day complication rate after cystectomy according to the Clavien–Dindo system [22]. Only complications ≥ grade 3 are included, apart from infections where all grades are reported. Data are presented for all patients and stratified according to characteristics at time of cystectomy (age, treatment period, NAC versus no NAC, smoking status, BMI), and according to weight change and GFR change for patients treated with NAC.

Characteristics	Any grade infections	Grade ≥ 3 all complications	Grade ≥ 3 infections	Grade ≥ 3 gastrointestinal	Grade ≥ 3 heart/circ	Grade ≥ 3 kidney failure	Grade ≥ 3 other
All patients, *N* = 124	55 (53)	34 (27)	23 (19)	10 (8.1)	7 (5.6)	4 (3.2)	4 (3.2)
Age							
< 75 y (*N* = 83)	38 (46)	19 (23)	14 (17)	4 (4.8)	4 (4.8)	1 (1.2)	2 (2.4)
≥ 75 y (*N* = 41)	28 (68)	15 (37)	9 (22)	6 (15)	3 (7.3)	3 (7.3)	2 (4.9)
Treatment period							
2011–2016	32 (48)	14 (21)	9 (13)	4 (6.0)	2 (3.0)	2 (3.0)	2 (3.0)
2017–2021	34 (60)	20 (35)	14 (25)	6 (10)	5 (8.8)	2 (3.5)	2 (3.5)
NAC status							
NAC	27 (46)	14 (24)	9 (15)	3 (5.1)	2 (3.4)	1 (1.7)	2 (3.4)
No NAC	39 (60)	20 (31)	14 (22)	7 (11)	5 (7.7)	3 (4.6)	2 (3.1)
Smoking status							
Ever smoker	52 (53)	25 (25)	18 (18)	7 (7.1)	6 (6.1)	3 (3.0)	1 (2.8)
Never smoker	14 (56)	9 (36)	5 (20)	3 (12)	1 (4.0)	1 (4.0)	1 (4.0)
BMI							
< 25 kg/m^2^	29 (45)	15 (19)	12 (19)	8 (12.5)	2 (3.1)	2 (3.1)	0
≥ 25 kg/m^2^	37 (62)	19 (32)	11 (18)	2 (3.3)	5 (8.3)	2 (3.3)	4 (6.7)
Weight change^a^							
Increase/stable	15 (42)	8 (22)	4 (11)	1 (2.8)	1 (2.8)	0	2 (5.6)
Decrease	12 (52)	6 (26)	5 (22)	2 (8.7)	1 (4.3)	1 (4.3)	0
GFR change^a^							
Stable/improved	15 (50)	9 (30)	6 (20)	3 (10)	0	1 (3.3)	1 (3.3)
Worsened	12 (41)	5 (17)	3 (10)	0	2 (6.9)	0	1 (3.4)

Abbreviations: NAC: neoadjuvant chemotherapy; BMI: body mass index; GFR: glomerular filtration rate; N: numbers; y: years.

Data are presented as numbers (%).

a Only for patients treated with minimum one cycle of neoadjuvant chemotherapy.

Thirty-four patients (27%) had a ≥ grade 3 complication (Table 2). Infections (19%) and gastrointestinal complications (8.1%) were the most common complications. No statistically significant differences were observed regarding incidence of all ≥ grade 3 complications (age *p* = 0.14; treatment period *p* = 0.11; NAC status *p* = 0.42; smoking status *p* = 0.32; BMI *p* = 0.32). Patients aged ≥ 75 years versus < 75 years had a borderline significantly higher incidence of ≥ grade 3 gastrointestinal complications (15% vs. 4.8%, *p* = 0.08). There were numerically fewer patients with any ≥ grade 3 complications among patients who received NAC than those without NAC, but none were statistically significant. Patients with a weight decrease after NAC had numerically higher incidences of all reported complications compared with patients with stable/increased weight, but none were statistically significant (data not shown).

Five patients died within 30 days after cystectomy (4.0%). One of these patients had received NAC, corresponding to a 30-day mortality rate at 1.7% after NAC, and 4.7% for patients without NAC.

### Relapse and survival

**Figure 3:**
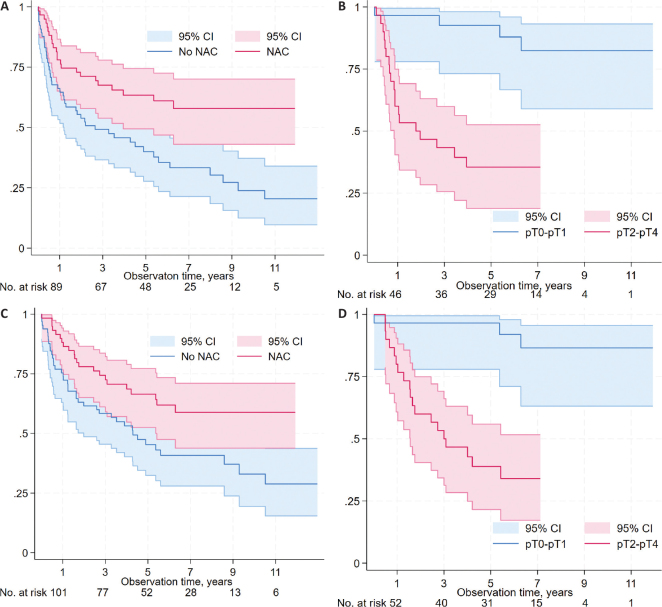
Relapse-free survival (RFS) and overall survival (OS) for all patients according to neoadjuvant chemotherapy (NAC) status (RFS: Figure 3A; OS: Figure 3C), and according to response to NAC (non-muscle invasive; ≤pT1 vs. muscle-invasive; ≥pT2) in the post-cystectomy specimen among NAC-treated patients (RFS: Figure 3B; OS: Figure 3D).

At the end of follow-up, 56 patients (45%) were alive and relapse-free (Table 3). The 5-year actuarial RFS was higher among patients treated with NAC than those without NAC (63% vs. 40%, *p* = 0.003, Figure 3A). Among NAC treated patients, the 5-year RFS was higher among patients who achieved ≤pT1 post-cystectomy compared to those with ≥pT2 (92% vs. 35%, *p* < 0.001, Figure 3B).

**Table 3:** Vital status at end of follow-up, 5-year RFS and 5-year OS, relapse site and causes of death according to NAC versus no NAC, and for all patients.

Characteristic	NAC *N* = 59	No NAC *N* = 65	Total *N* = 124
Vital status at end of follow-up			
Alive, relapse-free	36 (61)	20 (31)	56 (45)
Alive, with relapse	1 (1.7)	6 (9.2)	7 (5.6)
Relapse, overall	19 (32)	22 (34)	41 (33)
Dead	22 (37)	39 (60)	61 (49)
5-year RFS, % (95% CI)	63 (49–75)	40 (28–52)	51 (42–60)
5-year OS, % (95% CI)	67 (53–77)	45 (32–57)	55 (46–65)
Relapse site			
Regional lymph nodes	14 (24)	15 (23)	29 (23)
Lung metastases	7 (12)	9 (14)	16 (13)
Liver metastases	5 (8.5)	9 (14)	14 (11)
Bone	8 (14)	3 (4.6)	11 (8.9)
Other^a^	5 (8.5)	5 (7.7)	10 (8.1)
Cause of death			
Metastatic bladder cancer	18 (30)	16 (25)	34 (56)
Treatment-related complications	1 (1.7)	8 (12)	9 (15)
Infection, not treatment-related	2 (3.4)	7 (11)	9 (15)
Atherosclerotic disease	1 (1.7)	2 (3)	3 (5)
Other causes^b^	0	6 (10)	6 (15)

Data are presented as numbers (*n*) (%), unless otherwise specified.

RFS: relapse-free survival; OS: overall survival; NAC: neoadjuvant chemotherapy; CI: confidence interval.

a Other relapse sites: adrenal gland (*n* = 3), brain (*n* = 2), carcinomatosis (*n* = 2), pelvic muscle, spleen, penis.

b Other causes of death include kidney failure, prostate cancer, gastrointestinal bleeding, chronic obstructive pulmonary disease, unknown causes.

The 5-year actuarial OS rate was higher among patients treated with NAC than those without NAC (67% vs. 45%, *p* = 0.02, Figure 3C). Among NAC treated patients, the 5-year OS was higher among patients who had ≤pT1 post-cystectomy than those with ≥pT2 (94% vs. 39%, *p* < 0.001, Figure 3D).

In total 61 patients (49%) died. A larger proportion of patients died due to metastatic disease among patients treated with NAC versus no NAC (30% vs. 25% of all patients), while a larger proportion of patients died from treatment-related complications (12% vs. 1.7%) and causes other than MIBC-related (24% vs. 5%) among patients without NAC (*p* = 0.002).

Univariate Cox regression analyses showed that treatment during 2017–2021 (vs. 2011–2016) was associated with a lower risk of relapse or death (HR 0.59, 95% CI 0.35–0.99; HR 0.54, 95% CI 0.31–0.95; Table 4). Increasing pT stage at cystectomy was associated with an increased risk of relapse or death (*p* for trend < 0.001, both). Patients treated with NAC versus no NAC had about half the risk of relapse or death. In multivariate Cox regression, treatment during 2017–2021 was associated with lower risks of relapse and death, while increasing pT stage was associated with higher risks of relapse and death (*p* for trend < 0.001, both).

**Table 4:** Hazard ratios for progression-free survival and overall survival.

Variable	Relapse-free survival		Overall survival	
	Univariate HR (95% CI)	Multivariate HR (95% CI)	Univariate HR (95% CI)	Multivariate HR (95% CI)
Age at cystectomy	1.02 (0.99–1.04)	1.01 (0.98–1.03)	1.02 (0.99–1.05)	1.02 (0.99–1.05)
Treatment year				
2011–2016	1.00 (ref)	1.00 (ref)	1.00 (ref)	1.00 (ref)
2017–2021	0.59 (0.35–0.99)	0.53 (0.30–0.92)	0.54 (0.31–0.95)	0.45 (0.25–0.81)
pT stage, at cystectomy				
pT0/pTis/pT1	1.00 (ref)	1.00 (ref)	1.00 (ref)	1.00 (ref)
pT2	4.35 (1.86–10.2)	4.50 (1.82–11.1)	5.49 (2.07–14.6)	6.56 (2.36–18.3)
pT3/pT4	7.37 (3.28–16.6)	7.76 (3.22–18.7)	8.38 (3.27–21.5)	10.0 (3.64–27.6)
No NAC	1.00 (ref)	1.00 (ref)	1.00 (ref)	1.00 (ref)
NAC	0.48 (0.29–0.79)	1.07 (0.58–2.02)	0.54 (0.32–0.91)	1.41 (0.73–2.73)
Never smoker	1.00 (ref)		1.00 (ref)	
Ever smoker	0.79 (0.44–1.40)		0.79 (0.44–1.40)	
BMI < 25 kg/m^2^	1.00 (ref)		1.00 (ref)	
BMI ≥ 25 kg/m^2^	1.08 (0.67–1.74)		1.03 (0.62–1.70)	
eGFR ≥ 90 mL/min/1.73 m^2^	1.00 (ref)		1.00 (ref)	
eGFR 60–89	0.79 (0.39–1.59)		0.75 (0.36–1.58)	
eGFR < 60	1.41 (0.70–2.83)		1.31 (0.63–2.74)	

HR: hazard ratio; CI: confidence interval; p: pathologic; NAC: neoadjuvant chemotherapy; BMI: body mass index; eGFR: estimated glomerular filtration rate.

## Discussion

In this population-based study including urothelial MIBC patients treated with radical cystectomy during 2011–2021, the utilization of NAC was 48%. NAC treatment was not associated with any increased 30-day complication rate. The NAC treated patients who achieved a downstaging to ≤pT1 had impressive 5-year RFS and OS rates of 92% and 94%, respectively.

The ddMVAC regimen used at our institution was well-tolerated and with high rates of pathologic down-staging in two phase II studies [18, 19]. Furthermore, a higher complete response rate from ddMVAC compared to standard NAC was observed in a large retrospective trial among 824 patients 40% of whom received NAC [26]. The VESPER trial has recently reported improved 5-year OS rates with six cycles of ddMVAC compared with four cycles of cisplatin/gemcitabine in the neoadjuvant subgroup (66% vs. 57%) [5], suggesting ddMVAC as the standard NAC regimen.

The proportion receiving NAC herein (48%) is higher than that reported by more recent studies, with rates ranging from 16 to 44% [9, 13, 26–28]. The NAC rates seem to be high in countries with public health care systems providing equal access to treatments which is the case for Sweden and Norway, or with universal health insurance coverages as in Japan, with reported NAC rates up to 42 and 44% [9]. In addition, the NAC utility rate in our most recent treatment period 2017–2021 at 51% is in line with recently published national registry data from Denmark where it was reported that 213/425 (50%) of patients operated with cystectomy for MIBC received NAC [29]. Nonetheless, it is questionable whether the NAC utilization rate can be further increased above 50%, given the toxicity profile from cisplatin-based chemotherapy and the high median age at diagnosis (72 years herein). Supporting other studies, we found that age > 75 years and comorbidities, in particular cardiovascular disease and reduced kidney function, were the most common reasons for not being offered NAC, factors that also influence survival [10, 11].

Concern about increased rates of surgical complications post-cystectomy has been suggested as a possible reason for not offering NAC. However, the 30-day grade ≥ 3 Clavien–Dindo complication rates did not differ according to NAC status in our study, supporting findings from recent retrospective studies [13, 28, 30]. The 30-day mortality rate was low and numerically lower after NAC versus no NAC (1.7% vs. 4.7%). The numerically higher 30-day mortality rate among patients not treated with NAC probably reflects the fact that these patients had more comorbidity. The mortality rates reported herein corroborate numbers reported in the European guidelines [1], as well as data from a large Swedish study (90-day mortality rates at 2.7 and 6.3%, respectively) [13]. In light of concerns regarding surgical complications and loss of bladder function, other treatment options for MIBC are available, that is trimodality treatment with similar outcomes in retrospective analyses [31].

We observed that 49% of the patients treated with NAC achieved pathologic downstaging to ≤pT1, which is in line with the rates of 49–52% reported in the phase II trials [18, 19]. Some of this downstaging may also relate to a complete transurethral resection of the bladder. The complete response rate (pT0) herein was 29%, somewhat lower than numbers reported by three other studies with rates at 38–42% [18, 26, 32]. Two of these studies had, however, a higher number of ddMVAC cycles with 60% of patients treated with six cycles in the VESPER trial [32], and 91% received ≥ three cycles in the Peyton study [26]. In line with these studies, we have recently changed our treatment recommendation to four cycles of ddMVAC.

Our 5-year OS rate at 45% for patients without NAC is similar to the historically 5-year OS rate at 45% for these patients in a large meta-analysis [8], while our 5-year OS rate at 67% for patients treated with NAC is considerably better than previous results from meta-analyses with rates at 50 and 53% [4, 8]. Our 5-year OS rate after NAC, regardless of pathologic response, is in line with results from the VESPER trial, reporting a 5-year OS rate at 66% after ddMVAC [5].

Survival after NAC is strongly influenced by whether a pathologic downstaging is achieved. The observation time after NAC in our study is a median of 5.2 years, considerably longer than in comparable studies. Even so, our 5-year RFS rate among NAC treated patients with downstaging to ≤pT1 at 92% is comparable to the 1-year RFS at 89% and 2-year RFS at 88% reported in the phase II studies [18, 19], and our 5-year OS rate at 94% seems to be higher than the 2-year OS rate at 88% in another study [26]. On the other hand, patients who do not achieve a pathologic downstaging to non-MIBC after NAC seem to have a survival which is similar or even inferior to patients not treated with NAC. Consequently, there is a need for predictive biomarkers that can identify patients who will benefit from NAC, as well as new treatment options for non-responders. A move in the right direction is a recent study reporting that certain molecular subtypes of MIBC were more responsive to NAC [33].

Treatment after 2016 was associated with a 50% reduction of relapse or death compared with the earlier treatment period. The better results after 2016 might be explained by improved diagnostic procedures including standardized waiting times as a part of the national standardized cancer patient pathways [21], as well as improved surgical techniques with the implementation of robotic-assisted cystectomy in 2019 [20]. The improved OS is probably also influenced by better systemic treatment options for patients with relapse after cystectomy [1].

Strengths of this study include the population-based design, where all patients treated with radical cystectomy for urothelial MIBC in our region were included, and with a high degree of data completeness. The same chemotherapy regimen was applied during the whole study period. As a limitation, comorbidity was not systematically registered, but the treatment of NAC versus no NAC was strongly related to comorbidity. The low number of patients with ≥ grade 3 complications in the different categories introduced a possible type II bias, and prevented us from drawing any conclusions regarding risks of complications.

In conclusion, our NAC utility rate at 48% was high, and NAC was not associated with any increased rate of postoperative complications. Survival after NAC is strongly influenced by whether a pathologic downstaging is achieved, and there is a large need for predictive biomarkers to select patients who will benefit from NAC. Survival after a radical cystectomy also depends on the surgical technique applied, with promising results for en bloc radical cystectomy [34].

## Data Availability

The data are not available due to restrictions from the data protection officer and the Regional Committee for Medical and Research Ethics
